# Supplementary material for: Research on parameter identification of shaking table systems based on the RLS method
Source: PLoS One. 2022 Dec 28;17(12):e0279092. doi: 10.1371/journal.pone.0279092 (PMC9797076; doi:10.1371/journal.pone.0279092)
Supplement: Attachment — Submitted filename: PLOS ONE Review Comments.docx [file pone.0279092.s001.docx]

**Review Comments**

**General Comments:**

1. In abstract spelling of keywords need to be fix.
2. Number of keywords should be at least five.
3. In Figure-04, at few locations text is not readable so increase the font size.

**Conclusions and Recommendations:**

It is recommended to divide the conclusions into three paragraphs. In the first para, state about the problem of investigation and your methodology in the current study. In the second para, describe about results and in the last paragraph add some recommendations for future line of action.
